# Effect of Tinidazole on Norfloxacin Disposition

**Published:** 2017

**Authors:** Sally Aly Helmy, Mona Ibrahim El-Assal

**Affiliations:** a *Department of Pharmaceutics, Faculty of Pharmacy, Damanhour University, Damanhour, Egypt.*; b *Department of Clinical and Hospital Pharmacy, Faculty of Pharmacy, Taibah University, AL-Madinah AL-Munawarah, Kingdom of Saudi Arabia.*; c *Department of Pharmaceutical Technology, Faculty of Pharmaceutical sciences and pharmaceutical industries, Future University, Egypt.*

**Keywords:** Norfloxacin, Tinidazole, Pharmacokinetics, Drug-drug interactions, Fixed-dose combination

## Abstract

Co-administration of norfloxacin (NFX) and tinidazole (TNZ) has been used for the treatment of gastrointestinal and urinary tract infections. Concomitant oral administration of NFX with TNZ may affect NFX absorption and consequently its blood concentration and pharmacological effect. The present study was undertaken to investigate the effect of TNZ at the usual clinical dosage on the pharmacokinetics of NFX in healthy volunteers. This study was conducted as an open-label, randomized, two-way crossover experimental design. After an overnight fast, subjects were randomized to receive a single oral dose of NFX 400 mg alone and the fixed-dose combination (FDC) of NFX /TNZ 400 mg/600mg on two different occasions separated by 1 week washout period between treatments. Blood samples were collected up to 24 h postdose, and plasma was analyzed for NFX concentrations by using HPLC. The pharmacokinetic properties of NFX after FDC administration were compared with NFX administered alone. Twelve healthy subjects were enrolled (6 in each part), and all subjects completed the study. None of the participants showed any sign of adverse drug reactions during or after the completion of the study. The 90% confidence interval (CI) between NFX alone and when co-administered with TNZ indicated the presence of an interaction between NFX and TNZ, which would significantly increase the systemic rate and exposure of NFX absorption. The co-administration of TNZ with NFX increased the AUC and C_max_ of NFX significantly compared with administration of NFX alone. The AUC and C_max_ of NFX alone were 6.0 µg.hr/mL (2.3-9.8) and 0.87 µg/mL (0.4-1.6), respectively whereas the corresponding AUC and C_max_ values after administration of FDC were 7.1 µg.hr/mL (4.0-10.6) and 0.97 µg/mL (0.4-1.7), respectively. The respective geometric mean ratios of NFX for AUC and C_max_ with TNZ were 1.197 [90% CI, 0.941-1.522] and 1.087 (90% CI, 0.807 -1.463) compared with NFX alone. Both T_max_ and Ka of NFX showed a significant decrease after administration of the combination compared to administration of NFX alone. The peak plasma concentration reached at 1.3 h (0.6-2.4) and 1.9 h (0.4-4.4) after oral administration of FDC and NFX alone, respectively.

Both NFX and TNZ were well tolerated. The interaction of TNZ with fluroquinolones should be investigated to determine whether this interaction is limited to NFX or if other fluroquinolones have the same pharmacokinetic interactions. Further studies are necessary to determine the role of P-gp and other transporters on NFX disposition and pharmacokinetics. Additionally, the influence of TNZ on the physiological activity of GIT should be investigated.

## Introduction

Norfloxacin (NFX) is a broad-spectrum fluoroquinolone antibacterial agent. It has a potent activity against *Gram-positive* and *Gram-negative* bacteria, and a limited activity against *anaerobes* (1). NFX exerts broad-spectrum bactericidal effects *via* inhibition of the essential bacterial enzyme DNA gyrase, an enzyme essential for bacterial DNA replication (2). It is used in the treatment of a variety of urinary tract infections, uncomplicated gonococcal infection, and gastrointestinal infections (2). NFX has been identified as class IV drugs (according to a biopharmaceutics classification system) that are characterized by a low solubility and poor permeability (3). The oral bioavailability for NFX ranges from 35-40% and its absorption is not affected by concomitant food intake. The time to reach peak plasma concentration is ~1–2 h (4). Absorption is rapid following single doses of 200, 400, and 800 mg with mean peak plasma levels of 0.75, 1.5, and 2.41 µg/mL, respectively (4). The level of plasma protein binding is 10–15% (4). The average half-life is 3–4 h (4). Approximately 30% of the drug is metabolized into six different metabolites with minimal antimicrobial activity than parent compound (5). NFX is eliminated through metabolism, biliary excretion, and renal excretion (6). Renal excretion occurs by both glomerular filtration and tubular secretion as evidenced by the high rate of renal clearance (~275 mL/min) where, ~26–32% of an administered dose is excreted as unchanged drug by glomerular filtration and tubular secretion (6). Whereas, fecal recovery accounts for another 8.3–53.3% (mean 30%) of unchanged drug while ~30% of the drug is eliminated in urine and feces as metabolites (6, 7).

Concomitant oral administration of NFX with other drugs may affect its absorption and consequently its blood concentration and pharmacological effect. Few reports have been reported about the effect of various drugs on the pharmacokinetics of NFX (8-11). Co-administration of NFX with multivalent cation containing products decreased the absorption of NFX from the gastrointestinal tract, which might be hazardous during the treatment of serious infections (8). Milk or yoghurt decreases the extent of NFX absorption by 40% (12). Probenecid reduce the urinary excretion of NFX by inhibiting its tubular secretion (9, 11). Drugs which cause urine alkalinzation, such as sodium bicarbonate, carbonic anhydrase inhibitors and citrates, reduced the solubility of NFX and might increase the possibility of crystalluria (10, 11). The therapeutic fixed dose combination (FDC) of NFX plus tinidazole (TNZ) is thought to be beneficial and is a widely used approach that provides complementary mechanisms of action for the treatment of gastrointestinal infections caused by bacterial or amoebic infection, prostatitis and urinary tract infections due to susceptible uropathogens. Accordingly, studying the effect of co-administration of TNZ with NFX is beneficial and may affect the bioavailability of NFX. To the best of our knowledge, no studies have been reported about the effect of TNZ on the pharmacokinetics of NFX. Therefore, the present study was undertaken to investigate the effect of TNZ at the usual clinical dosage on the pharmacokinetics of NFX in healthy volunteers.

## Experimental

*Participants*

Healthy volunteers were recruited (through advertisements posted around the medical center) and assessed for inclusion in the study. Volunteers were selected randomly from a volunteer database and underwent a standardized screening procedure, to confirm their eligibility, 14 day before admission. Eligible volunteers were men with age between 18 to 33 years, with an ideal body wt of >60 kg (calculated {height (cm) -100} ×0.9) and a body mass index between 20 and 26 kg/m^2^ (calculated {wt (kg)/height (m^2^)}). After a physical examination, to exclude any abnormality of the cardiovascular, respiratory, abdominal, or central nervous system, BP, and heart rate (HR) were measured and electrocardiogram was conducted. A general examination of the subject was performed to exclude any illness or abnormality (e.g., anemia, cyanosis, clubbing, jaundice, and lymphadenopathy). Blood samples (10 mL) were collected for full blood count, BUN and electrolytes, liver function tests, renal function tests and random blood glucose. Serologic tests were conducted for the presence of hepatitis B surface antigen, hepatitis C virus antibody and HIV antibodies. Urine samples were also collected for microscopic examination analysis. Volunteers were admitted to the study after a review of pathology reports, medical history and making sure they met all the study inclusion and exclusion criteria. On completion of the study, the physical examination and clinical laboratory measurements were repeated. Blood samples were drawn from each subject at study end for assessment of all laboratory parameters as mentioned here, except for serologic tests, which were not redone. The volunteers were instructed to abstain from taking any medication for at least 14 day before and during the study period. They were also prohibited from consuming caffeinated beverages within 3 day of the first dosing administration and until completion of the study. The use of drugs or caffeinated beverages was identified by self-report and medical history taken by the study investigator. All volunteers gave their written informed consent to participate in the study after they had been well informed about the study objectives, methods, and possible risks.

Subjects were excluded if they had any condition such as a clinically significant abnormal physical exam, medical history, or laboratory studies; sitting systolic BP (SBP) of >140 or <100 mmHg, diastolic BP (DBP) > 90 or <60 mm Hg, or a pulse rate of > 95 or < 50 beats/min at screening; history of orthostatic hypotension; history of serious intolerance, allergy, or sensitivity to NFX or TNZ ; history of blood dyscrasias; history of alcohol, smoking, or drug abuse; donation of blood during the 8 week prior to the study or plans to donate blood during or within 8 week of completing the study; unable to tolerate vein puncture and multiple blood samplings; any surgical/medical condition that might alter the absorption, distribution, metabolism, or excretion of NFX; administration of any medication within 1 week before the start of the study; or canꞌt follow instructions, according to the investigatorꞌs opinion.

*Study design*

This study was carried out and monitored in accordance with the International Conference of Harmonization (ICH) guidance on general considerations for clinical trials (13). Twelve healthy male volunteers participated in the pharmacokinetic study under fasting condition. This study was conducted as an open-label, randomized two-way crossover study design with 1 week washout between treatments. 

Subjects were randomized to participate in the study, during which they received a single oral dose of the 2 treatments followed by 240 mL of water after an overnight fast. Participants continued fasting for another 4 h after drug administration. The study consisted of treatments: A (NFX 400 mg alone) and B (FDC NFX /TNZ 400 mg/600mg). The subjects remained in a comfortable recumbent position for up to 8 h after dosing and remained under medical observation throughout the study period. Before they were allowed to ambulate, they sat up with a dependent position for 1 min prior to standing up. The subjects remained in the clinical research unit until 48 h after dosing. All subjects completed the two treatment periods of the study. They received standardized meals 4 and 9 h after drug administration. All dietary, smoking, and drug/herbal product restrictions were maintained throughout the study period. After discharge, the physical examination and clinical laboratory measurements were repeated for the assessment of tolerability.

*Pharmacokinetic evaluation*

For the pharmacokinetic assessments, blood samples (5mL) were collected from an indwelling intravenous cannula inserted into the antecubital vein of the forearm of each volunteer before the drug administration (blank), then at 0.25, 0.5, 0.75, 1, 1.5, 2, 2.5, 3, 4, 6, 8, 10, 12 and 24 h after drug administration in heparinized tubes (a total of 15 blood samples). Blood samples were centrifuged and the plasma was harvested and transferred to polystyrene microcentrifuge tube and stored at −80 °C until assay for NFX using a validated high-performance liquid chromatography (HPLC) method (14).

*Tolerability and safety evaluation*

Tolerability was assessed by physical examination and verbally questioning subjects regarding their well-being and any feelings of discomfort. Tolerability was assessed based on changes in vital signs (temperature, seated BP, pulse and HR) and laboratory tests (hematology, biochemistry, liver function, and urinalysis). The vital signs were measured before dosing in each period and approximately every 4 h thereafter; laboratory tests were performed at the baseline and at the end of the study. In addition, a physician questioned the volunteers about any abnormal or unpleasant conditions that may indicate adverse events occurring during the study, addressed them as required, and recorded them on the appropriate form. This physician was not blinded to the treatment but had no involvement in the study.

*Bioanalytic Assessments*

The plasma level of NFX was measured using a validated HPLC-UV method (14). Briefly, the mobile phase comprised 20 mm sodium dihydrogen phosphate-2 hydrate (pH was adjusted to 3.5 by phosphoric acid) and acetonitrile (75:25, vol/vol). The elution was isocratic at ambient temperature with a flow rate of 1.5 mL/min. The separation was achieved using a Hypersil^®^ C_18_ column (250 mm × 4.6 mm, 5μm; Thermo Fisher Scientific, Waltham, MA). The effluent was monitored using a Shimadzu SPD-10AVP UV–VIS detector at 260 nm for NFX and the internal standard (gatifloxacin), and peak areas were integrated and calculated electronically using the Class-VP data analysis program (all Shimadzu Scientific Instruments, Kyoto, Japan). 

The calibration curves include all drug concentrations measured in clinical practice with within-day and between-day accuracies and precision were in accordance with (15). Calibration curves (n=10) were found to be linear over the entire concentration range of NFX (0.025-5 µg/mL), with a correlation coefficient *R*^2^ > 0.999 throughout the course of the assay for NFX. The lower limit of quantification (LLOQ) and limit of detection (LOD) were 0.025 and 0.01 µg/mL, respectively. The within-day and between-day coefficients of variance (%CV) were always within ± 11% in the entire range of the calibration curve (0.025-5 µg/mL). The within-day accuracy ranged between 99.7 and 106.3%, whereas the between-day accuracy ranged between 98.9 and 108.1%. The concentrations of the quality control samples were 2, 0.5, and 0.1 μg/mL, respectively. The absence of interference of NFX with TNZ was verified.

**Figure 1.**
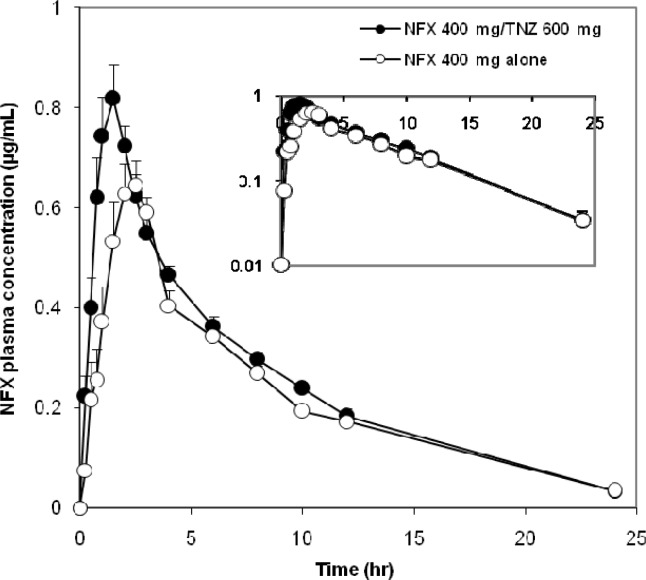
Mean linear and semilogarithmic NFX blood concentration-time profiles after oral administration of 400 mg dose to 12 healthy male volunteers (mean ± standard error

**Table 1:** Pharmacokinetic parameters of NFX after a single oral dose administration of 400 mg to 12 healthy male volunteers (mean ± SD).

**Parameters**	**NFX alone**	**FDC NFX/TNZ**	**90% Confidence Interval, Point Estimate** **(Lower Limit–Upper Limit)**	**P- value**
	**Mean ± SD**	**Mean ± SD**		
C_min_ (µg/mL)	0.026± 0.01	0.03± 0.01		
C_max_ (µg/mL)	0.87± 0.3	0.97 ± 0.4*	1.087 (0.807 -1.463)	0.032
t_max_ † (hr)	1.9 (0.4-4.4)	1.3 (0.6-2.4) *	0.775 (0.545-1.103)	0.040
AUC_0_ (µg.hr/mL)	6.0 ± 2.0	7.1± 1.9*	1.197 (0.941-1.522)	0.025
AUMC_0_ (µg.h^2^/mL)	44.0 ± 7.8	46.7± 5.7		
K_a_ (h^-1^)	1.3 ± 0.6	1.1 ± 0.37*	1.102 (0.836-1.451)	0.045
α (h^-1^)	1.34 ± 0.4	1.29 ± 0.5		
β (h^-1^)	0.089 ± 0.03	0.092 ± 0.05		
t _½_ of β (hr)	7.5 ± 2.0	8.8 ± 2.2		
K_12_ (h^-1^)	0.63 ± 0.3	0.61 ± 0.2		
K_21_ (h^-1^)	0.3 ± 0.1	0.36 ± 0.14		
MRT (hr)	8.8 ± 2.0	7.8 ± 1.2		
CL/F (L/hr)	71.5 ± 24.2	65.3 ± 17.3		
Vd/F (L)	302.4 ± 138.9	279.9 ± 126.7		

C_min_, minimum plasma concentration; C_max_, peak plasma concentration; t_max_, time to reach peak plasma concentration; AUC_0_ , area under the concentration-time curve from zero to infinity; AUMC_0_ , area under first moment curve from zero to infinity; K_a_, absorption rate constant; α and β, hybrid disposition rate constants; t _½_ of β, elimination half-life; K_12_, redistribution rate constant from the central to the peripheral compartment; K_21_, redistribution rate constant from the peripheral to the central compartment; MRT, mean residence time; CL/F, clearance; and V_d_/F, volume of distribution.

† Median (minimum to maximum).

* Statistically significant difference (*p* ≤ 0.05).

*Pharmacokinetic Analysis*

Pharmacokinetic parameters were calculated from actual sampling times and concentrations of plasma by a compartmental pharmacokinetic analysis. A two-compartment open pharmacokinetic model with first-order absorption and first-order elimination with or without lag time was utilized to describe the plasma concentration-time profile of NFX after oral administration. The plasma data were modeled using Win Nonlin version 2.0 pharmacokinetic software (Pharsight Corporation, 1994-1998, Palo Alto, CA). 

The area under the first moment of plasma drug concentration-time curve (AUMC) was calculated by trapezoidal integration and extrapolation to infinity. Mean residence time (MRT) was calculated as the ratio (AUMC)/ (AUC_0-∞_) (16).

*Statistical Analysis*

The sample size of this study was calculated based on the number estimated to provide > 80% power at a significance level of 0.05 (17). It was calculated using the intra-subject %CVs for AUC_0–∞_ and C_max_ parameters of 25% and 30%, respectively with an expected ratio between 0.90 and 1.05. All parameters were expressed as mean ± standard deviation (SD). Differences between treatments were assessed for FDC (T) versus NFX alone (R). Statistical comparisons between phases were made with one-way analysis of variance (ANOVA) model using the Minitab Statistical Package version 13 (Minitab, State College, PA) for crossover design (18). AUC_0-t_, AUC0_0–∞_, C_max_, and CL/F were evaluated after logarithmic transformation according to the international guidelines (19), providing point estimates and 90% confidential intervals (CI) (20) for the T/R ratio. A p-value of ≤0.05 and 90% CI fell outside the specified limit of 80% to 125%, were taken as the level of 

significance.

## Results

*Subjects*

The study enrolled 12 healthy volunteers; all of them completed the study and were included in the pharmacokinetic analyses. The mean (± SD) age of healthy volunteers was 28.1 (± 7.3) years, their mean body wt was 73.8 (± 8.6) kg, and their mean BMI was 22 (± 3.1) kg/m^2^. Safety profiles and pharmacokinetic characteristics were assessed in all 12 volunteers who completed the study. In the present study, measures were taken to protect the safety of the subjects, including prolonging the time of lying in bed and remaining in the clinical research unit, and enhancing the clinical monitoring and nursing. 

*Safety and Tolerability*

All enrolled volunteers were healthy, and none of the participants showed any signs of serious ADRs during or after completion of the study and no participants withdrew because of any ADRs. NFX and TNZ were well tolerated in the participated healthy male volunteers. The most commonly reported ADRs associated with NFX administration either alone or combined with TNZ were dizziness (1 report), Gastric distress (1 report), headache (2 reports). Physical examination, electrocardiograms, and laboratory tests revealed no clinically significant 

changes.

*Pharmacokinetic Characteristics*

The mean plasma concentration–time curves of NFX administered orally on different occasions as a single dose were represented in Figure 1. The Pharmacokinetics of NFX were best fitted to a two-compartment open model. The average PK parameters of NFX were summarized in Table 1**.** The bioavailability of NFX was increased when concomitantly administered with TNZ. The co-administration of NFX with TNZ significantly increased the systemic absorption represented by AUC (~15%) and the intensity of absorption represented by C_max_ (~11.5%) of NFX compared with the administration of NFX alone. The AUC and C_max_ of NFX alone were 6.0 µg.hr/mL (2.3-9.8) and 0.87 µg/mL (0.4-1.6), respectively whereas the corresponding AUC and C_max_ values after administration of FDC were 7.1 µg.hr/mL (4.0-10.6) and 0.97 µg/mL (0.4-1.7), respectively. The respective geometric mean ratios of NFX for AUC and C_max_ with TNZ were 1.197 [90% CI, 0.941-1.522] and 1.087 (90% CI, 0.807 -1.463) compared with NFX alone.

Concerning the rate of absorption, the T_max _and K_a_ of NFX showed a significant decrease after administration of the FDC compared to administration of NFX alone. The peak plasma concentration reached at 1.3 h. (0.6-2.4) and 1.9 h. (0.4-4.4) after oral administration of FDC and NFX alone, respectively. Furthermore, MRT of NFX was decreased by 13% when co-administered with TNZ compared with NFX alone.

## Discussion

Polypharmacy and complex treatment regimens have a detrimental effect on treatment compliance and adherence (21). A combination of NFX and TNZ is a preferred treatment option for treat respiratory, urinary and gastrointestinal tract infections in man and animals. The use of once-daily single-pill combination therapy of NFX plus TNZ is thought to be beneficial. This is due to their complementary modes of action in addition to once-daily dosing regimens, which are clearly preferred for patient compliance (21). With this background, the present study was undertaken to examine the influence of TNZ on the disposition profile of NFX in human after oral administration. The study design was more likely to identify any potential changes in pharmacokinetic parameters resulting from drug-drug interactions (DDIs). 

The effect of TNZ on pharmacokinetic parameters of NFX might be more reasonable to expect at steady state. However, due to practical limitations, the present pharmacokinetic study was conducted following a single dose administration. The disposition of NFX after a single oral dose (400 mg) was examined with or without combination with TNZ (600 mg). NFX was well tolerated by the volunteers; unexpected events that could have influenced the outcome of the study did not occur. All volunteers who started the study continued to the end and were discharged in good health. 

The pharmacokinetics of NFX when given alone were in good agreement with findings from previous investigations (22, 23), which emphasize the validity of the current results. NFX was rapidly absorbed after oral administration. Measurable concentrations (i.e., those above the LLOQ) were observed 15 to 30 min after dosing. Peak plasma levels were reached 0.4-4.4 h. after oral administration of NFX, and then declined with a terminal t_1/2_ ranging from 3 to 8.8 h The increased values of the terminal t_1/2_ could be attributed to the presence of a reduced glomerular filtration rate that increases the elimination half-life (24). Following oral administration of FDC, plasma concentration of NFX rapidly reached to peak concentration at 0.6-2.4 h and then decreased slowly reaching the last measurable plasma concentration of 0.03µg/mL after 24 h 

The 90% CIs were calculated for the difference between the log-transformed values of mean ratio (T/R) for NFX pharmacokinetic parameters estimated when it was administered alone and co-administered with TNZ (Table 1). The 90% CIs between NFX alone and when co-administered with TNZ indicated the presence of a DDI between NFX and TNZ. This DDI could significantly increase the bioavailability of NFX absorption. The increased bioavailability suggested that TNZ influenced both the rate and extent of absorption of NFX due to significant inhibition of metabolism. 

Drug absorption across the gastrointestinal tract is highly dependent on the affinity for membrane transporters as well as drug-metabolizing enzymes (25). Drug metabolism reactions are generally grouped into 2 phases. Phase I reactions involve changes such as oxidation, reduction, and hydrolysis and are primarily mediated by the cytochrome P450 (CYP) family of enzymes. Phase II reactions use an endogenous compound such as glucuronic acid, glutathione, or sulfate, to conjugate with the drug or its phase I–derived metabolite to produce a more polar end product that can be more readily excreted (26). Additionally, membrane transporters have a significant role in facilitating or preventing drug movement (27). P-glycoprotein (P-gp) in the intestinal epithelium plays an important role in the extrusion of many drugs from the blood into the intestinal lumen and in preventing drugs in the intestinal lumen from entering the bloodstream (28). Consequently, they are important in determining oral drug disposition by controlling absorption and bioavailability (29). 

NFX was extensively metabolized in the liver involving both Phase-I and Phase-II (30). Our results revealed that the values of AUC and C_max_ of NFX showed a significant increase following FDC administration. This might be attributed to the inhibition of enzymes mostly concerned with the hepatic metabolism of NFX that could enhance the systemic availability of NFX. Accordingly, the apparently decreased metabolism and/or excretion of NFX after co-administration with TNZ might be due to decreased expression of CYP2C9 and CYP3A4 in liver/intestine. TNZ is metabolized *via* the cytochrome P450 3A4 and 2C9, which might delay the excretion of NFX (31). Furthermore, TNZ is itself metabolized through hepatocytes as glucuronide conjugate (32). Therefore, the delay in NFX metabolism could be attributed to a competition between two substrates. After co-administration with TNZ, the plasma elimination half-life of NFX was increased (8.8 h). This might be related to inhibition of one or more enzyme(s) concerned with metabolism of NFX leading to its prolonged persistence in the body.

The observed increased absorption of NFX might be attributed to the ability of TNZ to influence drug transporter protein P-gp in the intestine. A decrease in P-gp-mediated exsorption into the intestines could, therefore, increase the absorption and oral bioavailability of NFX. A significant amount of NFX was excreted unchanged *via* renal mechanisms (33). Therefore, it could be hypothesized that TNZ might have delayed the excretory mechanism of NFX, since P-gp protein also exists in the proximal convoluted tubules (33).

In the present study, T_max_ exhibited a significant decrease, which might indicate that the co-administration of NFX and TNZ could slightly increase the rate of absorption for NFX due to the effect on organic anion transporters (OATs) or other transporters that could mediate their absorption. Therefore, additional studies are needed to gain more insight into the exact mechanism and the role of P-gp and other transporters in this interaction.

The extent of drug absorption through the biological membranes is generally affected by the physicochemical properties of a drug such as size, lipophilicity and charge (25). Accordingly, the effect of TNZ on the GIT permeability through modification of its physiological activity by opening of tight junctions and disrupting the membrane bilayer could not be ruled out and might have contributed to the improved absorption of NFX. This, however, requires further experimental evidence.

All enrolled volunteers were healthy, and none of them exhibited any signs of ADRs during or after completion of the study. Therefore, further multiple dose administration and long-term studies are needed to evaluate the clinical efficacy and the pharmacologic drug interactions of the combination therapy of NFX and TNZ. These studies are necessary to discover whether the observed DDI has any impact on the combined use of NFX and TNZ. The interaction of TNZ with fluroquinolones should be investigated to determine whether this interaction is limited to NFX or if other fluroquinolones have the same pharmacokinetic interactions.

## Conclusion

Both NFX and TNZ were well tolerated in this population of healthy male subjects. The findings from the current single-dose studies suggested that there was the evidence of significant drug interactions between NFX and TNZ among this volunteer study cohort. The co-administration of TNZ significantly increased the rate and extent of NFX absorption. Further studies are necessary to determine the role of P-gp and other transporters on NFX disposition. Additionally, the influence of TNZ on the physiological activity of GIT should be investigated.
